# Surgery Versus Radiation for Stage 1A NSCLC in Nonagenarians: 20 Years of Data, Decisions, and Outcomes

**DOI:** 10.1245/s10434-026-19551-y

**Published:** 2026-04-01

**Authors:** Niranjna Swaminathan, Rongbing Xie, Srivatsvan Swaminathan, James M. Donahue, Benjamin Wei

**Affiliations:** 1 Division of Cardiothoracic Surgery, UAB Department of Surgery, University of Alabama-Birmingham Medical Center, Birmingham, AL USA; 2 Heersink School of Medicine, The University of Alabama, Birmingham, AL USA; 3 Birmingham VA Medical Center, Birmingham, AL USA

**Keywords:** Nonagenarians, Early stage, NSCLS, Surgery, Radiation

## Abstract

**Background and Purpose:**

The optimal treatment strategy for non-small-cell lung cancer (NSCLC) in patients aged > 90 years is unclear. This study evaluates overall survival and treatment-specific outcomes in nonagenarians with stage 1A NSCLC.

**Methods:**

We retrospectively analyzed 2785 nonagenarians with stage 1A NSCLC from 2004 to 2024 using the National Cancer Database. Propensity score matching was performed using stage, tumor site, tumor characteristics, demographics, facility type and Charlson–Deyo comorbidity score. Overall survival was compared using Kaplan–Meier analysis.

**Results:**

Of 2785 patients, 707 (25%) received no treatment, 269 (13%) underwent surgery, and 1717 (83%) received radiation only. Patients undergoing surgery had the highest overall survival rate: 86.9% vs 82.8% vs 61.8% at 1 year (*p* < 0.05) and 38.3% vs 23.2% vs 12.0% at 5 years for surgery, radiation, and no treatment, respectively (*p* < 0.05). After propensity score matching, surgery remained associated with improved overall survival, with 3-year survival of 64.6% vs 44.0% (*p* < 0.05) and 5-year survival of 37.8% vs 20.7% for surgery and radiation, respectively (*p* < 0.05). Within the surgery group, 30- and 90-day mortality rates were 6% and 9%, respectively, the unplanned readmission rate was 6%, and the median hospital stay was 4 days. These rates were consistent across different Charlson–Deyo comorbidity score groups, and survival outcomes in the surgery cohort were not influenced by the facility type (*p* = 0.20).

**Conclusion:**

Surgical resection was associated with improved observed overall survival compared with radiation therapy among carefully selected patients, suggesting that chronological age alone should not preclude consideration of surgical evaluation.

**Supplementary Information:**

The online version contains supplementary material available at 10.1245/s10434-026-19551-y.

Non-small-cell lung cancer (NSCLC) accounts for approximately 81% of all lung cancer diagnoses and remains the leading cause of cancer-related mortality worldwide.^1^ More than half of NSCLC cases occur in adults aged ≥ 70 years, yet patients in their tenth decade of life (nonagenarians) are rarely represented in clinical trials.^2,3^

Advances in public health, medical care, and socioeconomic conditions have markedly increased life expectancy across the United States, creating a growing population of very elderly individuals at risk for malignancies such as early-stage NSCLC. Adults aged ≥ 65 years now account for nearly 30% of the total US population, and the number of individuals aged ≥ 90 years is projected to increase from approximately 1.9 million in 2020 to over 7.6 million by 2050.^1^ According to the 2022 Social Security Administration life table, a 90-year-old man in the USA can expect to live an additional 4.62 years, and a 90-year-old woman an additional 4.27 years on average.^2^ Despite advanced age, nonagenarians still can expect multiple years of life, thereby underscoring the relevance of curative or high-impact treatments in this demographic.

Previous population-based studies have reported associations between surgical resection and improved overall survival (OS) compared with non-surgical modalities for early-stage NSCLC. Multiple Surveillance, Epidemiology, and End Results (SEER) analyses have shown that surgery alone confers superior OS, with 2- and 5-year survival rates of 74% versus 63% and 61% versus 18%, respectively, when compared with radiation therapy.^3–5^ Similarly, another study reported a 5-year OS of 60% among operable patients compared with 3% in untreated cohorts.^6^ A recent study focusing exclusively on nonagenarians reaffirmed these findings, showing that surgery remains superior to stereotactic body radiation therapy (SBRT), chemoradiation, and chemotherapy in early-stage disease.^7^

Nonetheless, the adoption of SBRT has expanded rapidly, particularly among elderly and medically inoperable patients.^8–10^ However, there is limited level I evidence directly comparing SBRT and surgery in nonagenarians, leaving the optimal management strategy uncertain. Moreover, no population-based study has evaluated annual improvements in local therapy outcomes following the widespread implementation of SBRT in this unique cohort.

Given the growing nonagenarian population and evolving treatment landscape, this study aimed to compare survival outcomes between surgery and radiation therapy in nonagenarians with stage 1A NSCLC, using 20 years of national data to inform evidence-based treatment decisions in this increasingly prevalent and high-risk demographic.

## Methods

We conducted a retrospective cohort study using the National Cancer Database (NCDB), which captures approximately 70% of all newly diagnosed malignancies across the USA. The NCDB is jointly sponsored by the American College of Surgeons and the American Cancer Society and includes detailed demographic, clinical, and treatment variables collected from Commission on Cancer–accredited facilities. The study was deemed exempt from institutional review board oversight as the NCDB contains de-identified patient data.

**Fig. 1:**
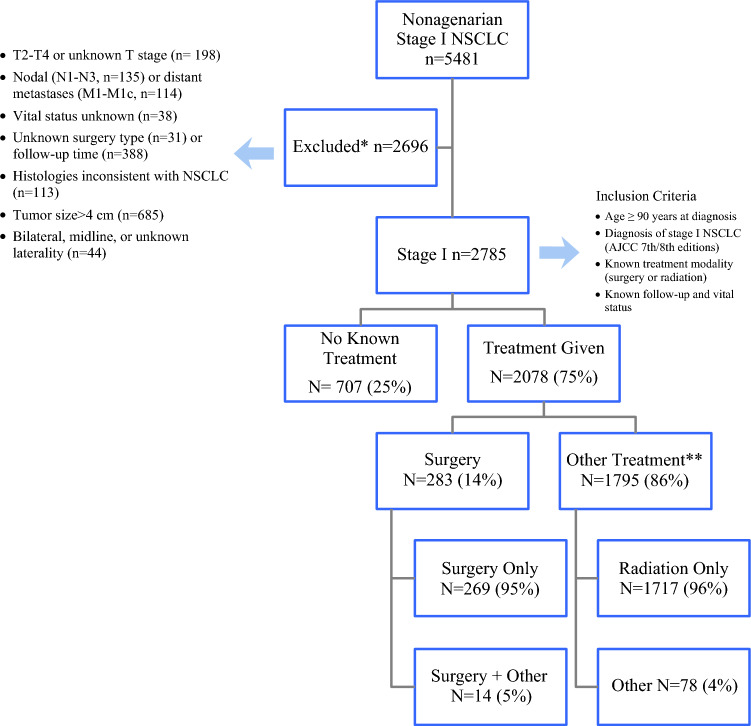
Cohort selection and treatment classification of nonagenarian patients (age ≥ 90 years) with stage 1A non-small-cell lung cancer (NSCLC). AJCC, American Joint Committee on Cancer

Patients aged ≥ 90 years at the time of diagnosis with stage 1A NSCLC as defined by the American Joint Committee on Cancer 7th or 8th edition staging system between 2004 and 2024 were included in the study. Eligible patients had documented treatment information (either surgery, radiation, or no treatment) and known follow-up and vital status. Patients were excluded if they had T2–T4 or unknown T stage (*n* = 2198), nodal disease (N1–N3, *n* = 135), or distant metastases (M1–M1c, *n* = 114). Additional exclusions included unknown vital status (*n* = 388), unknown surgery type (*n* = 31), missing follow-up time (*n* = 388), histologies inconsistent with NSCLC (*n* = 113), tumor size >4 cm (*n* = 685), and bilateral, midline, or unknown laterality (*n* = 44). In addition, patients who received multimodality treatment, including surgery plus radiation, surgery plus chemotherapy, or radiation plus chemotherapy, were excluded from the analytic cohort, as the study aimed to compare outcomes of single-modality definitive local therapy. After applying all criteria, the final cohort consisted of 2785 nonagenarian patients with stage 1A NSCLC (Fig. 1).

These patients were then categorized into three mutually exclusive treatment groups: surgery only, radiation only, and no definitive local therapy, defined as receipt of neither surgery nor radiation. Subgroup analyses were conducted for type of surgical resection (wedge vs segmentectomy vs lobectomy), Charlson–Deyo comorbidity score (CDS), and facility type. Demographic variables included age, sex, race/ethnicity, insurance status, median household income, and educational attainment at the ZIP-code level. Clinical variables included CDS (0, 1, 2, ≥3), tumor grade, T-stage (T1a–T1c), histologic subtype (adenocarcinoma, squamous, large cell, or other), and laterality. Facility-level characteristics included program type (academic/research, comprehensive community, community, or integrated network) and distance from residence to treating facility.

The primary outcome was OS from the date of diagnosis to death or last follow-up. Secondary outcomes included 30- and 90-day postoperative mortality, length of stay, unplanned readmission within 30 days, and margin status among surgically treated patients. Survival estimates were derived using Kaplan–Meier methods.

Baseline characteristics were compared between treatment groups using chi-squared or Wilcoxon rank-sum tests as appropriate. Logistic regression was used to identify predictors of surgical treatment. Covariates included in the multivariable model were sex, race, CDS, T-stage, tumor grade, histology, income, education, insurance, facility type, and year of diagnosis. Model fit was evaluated using the likelihood ratio and Wald chi-squared tests. Odds ratios (ORs) and 95% confidence intervals (CIs) were reported. To account for baseline differences, propensity score matching (PSM) was performed in a 1:1 ratio between surgery and radiation-only patients using all covariates listed in Table 1. Post-matching balance was verified using standardized mean differences (< 0.1 indicating adequate balance). Matched cohorts were compared using Kaplan–Meier survival analysis and log-rank tests, with subgroup analyses by comorbidity score and facility type. All analyses were performed using SAS 9.4 (SAS Institute, Cary, NC, USA). A two-tailed *p* < 0.05 was considered statistically significant.

**Table 1:** Baseline characteristics of patients undergoing surgery vs radiation (before and after propensity score matching [PSM])

Characteristics	Before PSM				After PSM			
	Overall *N* = 1986	Surgery *N* = 269	Radiation only *N* = 1717	*P*-value	Overall *N* = 502	Surgery *N* = 251	Radiation only *N* = 251	*P*-value
*Sex*				0.91				0.79
Male	959 (48)	129 (48)	830 (48)		241 (48)	119 (47)	122 (49)	
Female	1027 (52)	140 (52)	887 (52)		261 (52)	132 (53)	129 (51)	
*Race*				0.66				0.77
Black	88 (4)	13 (5)	75 (4)		19 (4)	11 (4)	8 (3)	
White	1840 (93)	246 (91)	1594 (93)		464 (92)	231 (92)	233 (93)	
Other/unknown	58 (3)	10 (4)	48 (3)		19 (4)	9 (4)	10 (4)	
*Total Charlson-Deyo Score*				0.13				0.23
0	1294 (65)	171 (64)	1123 (65)		330 (66)	161 (64)	169 (67)	
1	392 (20)	64 (24)	328 (19)		97 (19)	57 (23)	40 (16)	
2	170 (9)	23 (9)	147 (9)		51 (10)	22 (9)	29 (12)	
3 or more	130 (7)	11 (4)	119 (7)		24 (5)	11 (4)	13 (5)	
*T Stage*				<.0001				0.28
T1a	465 (23)	67 (25)	398 (23)		112 (22)	64 (26)	48 (19)	
T1b	785 (40)	77 (29)	708 (41)		164 (33)	76 (30)	88 (35)	
T1c	348 (18)	34 (13)	314 (18)		64 (13)	34 (14)	30 (12)	
T1 NOS (not otherwise specified)^a^	388 (20)	91 (34)	297 (17)		162 (32)	77 (31)	85 (34)	
*Laterality*				0.79				0.65
Right	1122 (57)	150 (56)	972 (57)		277 (55)	136 (54)	141 (56)	
Left	864 (44)	119 (44)	745 (43)		225 (45)	115 (46)	110 (44)	
*Primary site*				0.42				0.34
Upper lobe	1214 (61)	165 (61)	1049 (61)		306 (61)	152 (61)	154 (61)	
Middle lobe	70 (4)	13 (5)	57 (3)		21 (4)	13 (5)	8 (3)	
Lower lobe	654 (33)	88 (33)	566 (33)		163 (32)	83 (33)	80 (32)	
Overlapping lesion	5 (0)	0 (0)	5 (0)		1 (0)	0 (0)	1 (0)	
Primary site unknown	43 (2)	3 (1)	40 (2)		11 (2)	3 (1)	8 (3)	
*Histology*				0.02				0.96
Squamous	492 (25)	61 (23)	431 (25)		120 (24)	59 (24)	61 (24)	
Adenocarcinoma	1224 (62)	170 (63)	1054 (61)		313 (62)	156 (62)	157 (63)	
Large cell	8 (0)	4 (1)	4 (0)		5 (1)	3 (1)	2 (1)	
Other	262 (13)	34 (13)	228 (13)		64 (13)	33 (13)	31 (12)	
*Grade*				<.0001				0.97
1	217 (11)	40 (15)	177 (10)		75 (15)	39 (16)	36 (14)	
2	379 (19)	92 (34)	287 (17)		160 (32)	81 (32)	79 (31)	
3	297 (15)	69 (26)	228 (13)		124 (25)	63 (25)	61 (24)	
4	4 (0)	1 (0)	3 (0)		2 (0)	1 (0)	1 (0)	
*Income*				0.04				0.80
<$46,277	210 (11)	23 (9)	187 (11)		50 (10)	23 (9)	27 (11)	
$46,277–57,856	369 (19)	39 (15)	330 (19)		82 (16)	37 (15)	45 (18)	
$57,857–74,062	415 (21)	62 (23)	353 (21)		121 (24)	61 (24)	60 (24)	
$74,063 or more	720 (36)	115 (43)	605 (35)		194 (39)	102 (41)	92 (37)	
Not available	272 (14)	30 (11)	242 (14)		55 (11)	28 (11)	27 (11)	
*Education*				0.52				0.99
15.3% +	241 (12)	38 (14)	203 (12)		73 (15)	35 (14)	38 (15)	
9.1–15.2%	426 (21)	53 (20)	373 (22)		99 (20)	49 (20)	50 (20)	
5.0–9.0%	555 (28)	81 (30)	474 (28)		153 (30)	76 (30)	77 (31)	
5.0%	496 (25)	67 (25)	429 (25)		122 (24)	63 (25)	59 (24)	
Not available	268 (13)	30 (11)	238 (14)		55 (11)	28 (11)	27 (11)	
*Insurance*				0.21				0.69
Not insured	3 (0)	1 (0)	2 (0)		2 (0)	1 (0)	1 (0)	
Private	109 (5)	20 (7)	89 (5)		33 (7)	20 (8)	13 (5)	
Medicaid	11 (1)	2 (1)	9 (1)		5 (1)	2 (1)	3 (1)	
Medicare	1814 (91)	244 (91)	1570 (91)		455 (91)	226 (90)	229 (91)	
Other government	33 (2)	1 (0)	32 (2)		4 (1)	1 (0)	3 (1)	
Unknown	16 (1)	1 (0)	15 (1)		3 (1)	1 (0)	2 (1)	
*Facility type*				<0.001				0.56
Community	114 (6)	16 (6)	98 (6)		32 (6)	16 (6)	16 (6)	
Comprehensive	823 (41)	95 (35)	728 (42)		171 (34)	93 (37)	78 (31)	
Academic/research	630 (32)	114 (42)	516 (30)		206 (41)	98 (39)	108 (43)	
Integrated network	419 (21)	44 (16)	375 (22)		93 (19)	44 (18)	49 (20)	
*Distance from Facility (per mile)*	7.5 (3.6–17.7)	7.8 (3.3–19.9)	7.5 (3.6–17.4)	0.99	9.1 (3.8–24.2)	9.1 (3.6–25.7)	9.1 (4.6–22.9)	0.93

Data are presented as *n* (%) or median (interquartile range) unless otherwise indicated

^a^T1 NOS (not otherwise specified) indicates tumors coded as T1 without subclassification in the National Cancer Database dataset

## Results

Of the 2785 eligible patients, 2078 (75%) received definitive local therapy and 707 (25%) received no definitive local therapy. Of patients receiving definitive local therapy, 269 (13%) underwent surgery and 1717 (83%) received radiation. Surgical patients were more often treated at academic/research programs than at non-academic/research programs (42% vs 30%, *p* < 0.001), had a higher prevalence of grade 1–2 tumors (49% vs 27%, *p* < 0.001), and were more frequently in higher income quartiles (43% vs 35%, *p* = 0.04). Demographics (sex, race) and comorbidity burden were similar between groups (*p* > 0.10).

After 1:1 propensity score matching (*n* = 502: 251 surgery vs 251 radiation), all covariates were balanced (standardized differences < 0.10). Post-matching, covariates including stage, grade, histology, facility type, and socioeconomic indicators were well balanced between groups (all *p* > 0.05) [Table 1]. In the adjusted Cox model, significant type III associations were observed for histology, income quartile, and year of diagnosis. Compared with adenocarcinoma, squamous histology was associated with worse survival (hazard ratio 1.33; 95% CI 1.00–1.76; *p* = 0.05). Income demonstrated a protective association as patients in the highest income quartile had a 49% lower mortality hazard than the lowest quartile (hazard ratio 0.51; 95% CI 0.31–0.82; *p* = 0.004). Although grade was significant overall, no individual grade level differed significantly from grade 1 in pairwise contrasts, suggesting heterogeneity across categories (Table 2). In contrast, sex, race, CDS, T stage, laterality, and primary site were not significantly associated with survival (all *p* > 0.10).

**Table 2:** Multivariable Cox proportional hazards model among propensity-scored matched nonagenarian patients (aged ≥ 90 years) with stage 1A non-small-cell lung cancer

Variable (reference)	Level	HR (95% CI)	*p*-Value
Radiation only vs surgery		1.63 (1.30–2.05)	<0.0001
Male vs female		1.43 (1.13–1.80)	0.0033
Race (ref=white)	Black	0.78 (0.39–1.54)	0.4631
	Other/unknown	0.41 (0.19–0.87)	0.0192
Charlson-Deyo (ref=0)	1	1.34 (1.01–1.78)	0.0443
	2	1.02 (0.70–1.49)	0.9077
	≥3	1.90 (1.13–3.18)	0.0145
T stage (ref=T1a)	T1a	0.78 (0.53–1.13)	0.1960
	T1b	0.95 (0.65–1.38)	0.7850
	T1c	1.10 (0.60–2.01)	0.7525
Laterality (ref=right)	Left	0.91 (0.72–1.14)	0.4017
Primary site (ref=upper lobe)	Middle lobe	0.86 (0.48–1.52)	0.5968
	Lower lobe	1.02 (0.80–1.31)	0.8535
	Overlapping lesion	1.33 (0.15–11.84)	0.7999
	Unknown	0.66 (0.29–1.51)	0.3331
Histology (ref=adenocarcinoma)	Large cell	2.98 (0.50–17.74)	0.2317
	Other	1.32 (0.91–1.90)	0.1511
	Squamous	1.33 (1.00–1.76)	0.0504
Grade (ref=1)	2	1.10 (0.77–1.58)	0.5918
	3	1.08 (0.73–1.59)	0.7093
	4	0.33 (0.03–3.62)	0.3542
	9	0.88 (0.59–1.32)	0.5409
Income (ref=< $46,277)	$46,277–$57,856	0.60 (0.38–0.96)	0.0318
	$57,857–$74,062	0.56 (0.36–0.89)	0.0136
	≥$74,063	0.51 (0.31–0.82)	0.0048
	Not available	0.55 (0.30–1.00)	0.0521
Education (ref=<5.0%)	≥15.3%	0.80 (0.51–1.25)	0.3263
	5.0–9.0%	0.87 (0.63–1.19)	0.3784
	9.1–15.2%	0.95 (0.62–1.44)	0.7958
Insurance (ref=private)	Unknown	0.30 (0.05–1.83)	0.2044
	Medicaid	2.02 (0.68–6.03)	0.2024
	Medicare	1.10 (0.68–1.79)	0.6955
	Not insured	28.98 (5.36–156.69)	0.0001
	Other government	2.18 (0.47–10.11)	0.3176
Facility type (ref=academic/research)	Community	1.45 (0.87–2.40)	0.1539
	Comprehensive community	1.01 (0.77–1.33)	0.9373
	Integrated network	0.99 (0.72–1.38)	0.9661
Year of diagnosis (ref=2021)	2004	1.19 (0.42–3.35)	0.7436
	2005	1.15 (0.44–3.01)	0.7773
	2006	1.28 (0.46–3.50)	0.6404
	2007	1.56 (0.58–4.19)	0.3811
	2008	1.59 (0.64–3.97)	0.3159
	2009	1.75 (0.66–4.60)	0.2599
	2010	1.51 (0.67–3.40)	0.3216
	2011	1.25 (0.56–2.77)	0.5929
	2012	1.36 (0.57–3.25)	0.4858
	2013	0.97 (0.39–2.39)	0.9429
	2014	1.15 (0.50–2.63)	0.7439
	2015	1.24 (0.52–2.96)	0.6330
	2016	1.41 (0.58–3.41)	0.4521
	2017	1.31 (0.54–3.19)	0.5457
	2018	0.95 (0.44–2.03)	0.8906
	2019	1.82 (0.80–4.14)	0.1523
	2020	0.86 (0.36–2.02)	0.7182

CI, confidence interval; HR, hazard ratio

Among the 269 surgical patients, wedge resection was performed in 124 patients (46%), segmentectomy in 49 (18%), lobectomy in 91 (34%), and other procedures in five (2%). Among cases with a documented operative approach (*n* = 209), 79 procedures (38%) were thoracoscopic, 39 (19%) were robotic-assisted, 75 (36%) were open, and four (2%) were converted to open.

### Overall Survival

**Fig. 2:**
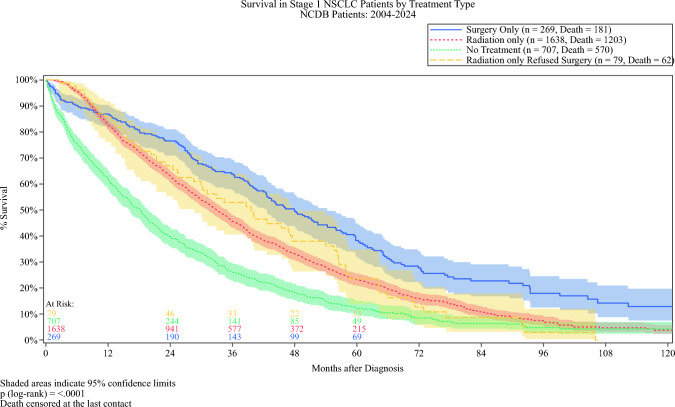
Overall survival among nonagenarian patients (aged ≥ 90 years) with stage 1A non-small-cell lung cancer (NSCLC) by treatment type before propensity score matching (PSM). Surgery (solid blue line) was associated with significantly higher survival than was radiation (dotted red line), radiation after surgical refusal (dashed yellow line), and no treatment (solid green line) (log-rank *p* < 0.0001). Shaded regions indicate 95% confidence intervals. Median follow-up 39 months (interquartile range 12–78). NCDB, National Cancer Database

The median follow-up was 29 months (interquartile range [IQR] 15–48) overall, 38 months (IQR 21–61) for the surgery group, and 28 months (IQR 14–46) for the radiation group. Surgical resection was associated with significantly higher observed OS than radiation therapy. In the unmatched cohort, 1-, 3-, 5-, and 10-year OS rates for the surgery group were 86.9%, 63.9%, 38.3%, and 12.8%, respectively, versus 82.7%, 45.6%, 23.2%, and 3.8% for the radiation group (log-rank* p* < 0.001). Patients who received no definitive local therapy had substantially lower survival across all intervals, with a 5-year OS of 12.0%, whereas those who refused surgery but received radiation had a 5-year OS matching that of the patients receiving radiotherapy at 23.5%. Both surgery and radiation were associated with improved long-term survival compared with observation, though surgery conferred the greatest benefit (Fig. 2).

**Fig. 3:**
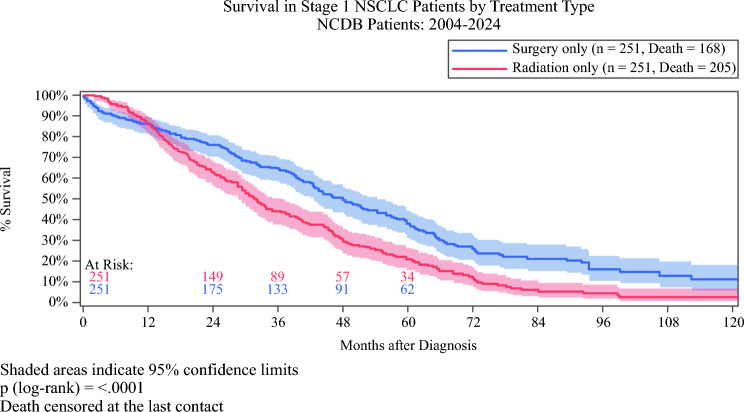
Kaplan–Meier curves comparing overall survival for matched surgery vs radiation cohorts. NCDB, National Cancer Database; NSCLC, non-small-cell lung cancer

In the propensity-matched analysis (*n* = 502; 251 surgery vs 251 radiation), surgery continued to be associated with improved OS. Post-matching, 1-, 3-, 5-, and 10-year OS rates were 86.4%, 64.6%, 37.8%, and 11.1% for the surgical cohort, compared with 86.4%, 44.0%, 20.7%, and 2.6% for the radiation cohort (log-rank *p* < 0.001) [Fig. 3].

**Fig. 4:**
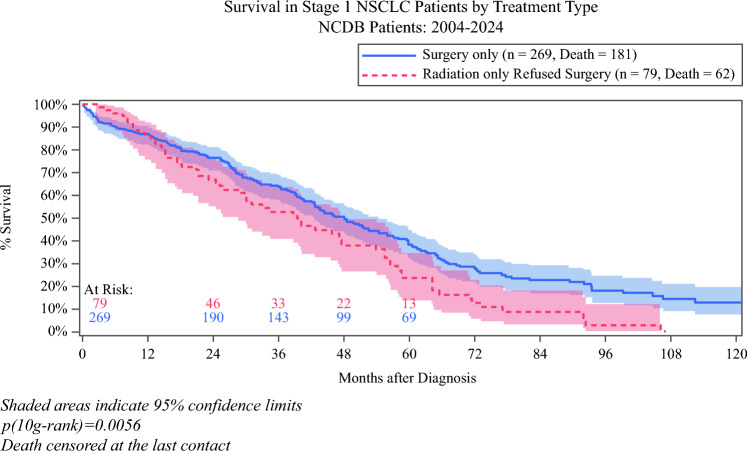
Overall survival among nonagenarian patients (aged ≥ 90 years) with stage 1A non-small-cell lung cancer (NSCLC) who were surgical candidates but received radiation (*n* = 79) compared with those undergoing surgery (*n* = 269) before propensity score matching. NCDB, National Cancer Database

When evaluating patients clinically eligible for surgery who refused operative intervention and instead received radiation (*n* = 79), OS was significantly worse than for those who underwent surgery (*n* = 269). Early survival was comparable between groups (1-year OS 100% vs 97.4%, and 3-year OS 98.7% vs 92.5% for radiation vs surgery). However, substantial differences emerged over time: by 5 years, OS was 23.5% for radiation compared with 38.3% for surgery, and by 10 years, 5.7% vs 12.8%, respectively (log-rank *p* = 0.0056) (Fig. 4). Propensity matching was attempted for this subgroup but was not feasible, as only 12 patients who received radiation could be matched, yielding unstable and uninterpretable survival estimates.

### Subgroup Analysis

Among patients who underwent surgical resection, wedge resection was the most common procedure (*n* = 124 [46.1%]), followed by lobectomy (*n* = 91 [33.8%]) and segmentectomy (*n* = 49 [18.2%]). Anatomic resection was associated with more favorable long-term survival than wedge resection (5-year OS of 44.0% for lobectomy, 46.4% for segmentectomy, 30.4% for wedge resection, *p* = 0.03) [Supplementary Fig. 1].

When stratified by CDS, OS differed across comorbidity levels in both the surgical and the radiation cohorts. Among surgical patients, 1-, 3-, 5-, and 10-year OS rates for CDS = 0 were 87.0%, 67.1%, 45.6%, and 16.6%, respectively. Corresponding 5-year OS rates for CDS = 1, CDS = 2, and CDS ≥3 were 28.1%, 21.2%, and 21.8%, respectively (Supplementary Fig. 2). Among radiation-treated patients, 1-, 3-, 5-, and 10-year OS rates for CDS = 0 were 85.0%, 49.5%, 24.6%, and 3.3%, respectively. Corresponding 5-year OS rates for CDS = 1, CDS = 2, and CDS ≥ 3 were 21.7%, 23.3%, and 13.0%, respectively (Supplementary Fig. 3).

No statistically significant differences in OS were seen within surgical and radiation groups when patients were stratified by facility type (*p* = 0.20 and *p* = 0.11, respectively) [Supplementary Figs. 4 and 5].

### Postoperative Outcomes

Among nonagenarian patients undergoing surgical resection, the median length of stay was 4 days (IQR 2–7). In total, 30-day readmission occurred in 6% of patients, including 6% unplanned and 2% planned readmissions. The 30-day and 90-day postoperative mortality rates were 6% and 9%, respectively. When stratified by CDS, there were no significant differences between patients with CDS = 0 and CDS > 0 in 30-day readmission (*p* = 0.17), 30-day mortality (*p* = 0.49), or 90-day mortality (*p* = 0.84) [Table 3].

**Table 3:** Postoperative outcomes stratified by Charlson–Deyo Score (CDS) among nonagenarian patients undergoing surgical resection for stage 1A non-small-cell lung cancer

Postoperative outcome	Overall	CDS=0	CDS>0	*P*-value
Length of stay, days	4 (2–7)	4 (2–7)	4 (2–7)	0.62
30-day readmission				0.17
No readmission	242 (90)	154 (90)	88 (90)	
Unplanned readmission	17 (6)	9 (5)	8 (8)	
Planned readmission	6 (2)	0	6 (4)	
30-day mortality	15 (6)	11 (6)	4 (4)	0.49
90-day mortality	23 (9)	15 (9)	8 (8)	0.84

Data are presented as median (interquartile range) or *n* (%) unless otherwise indicated

## Discussion

In this national analysis of nonagenarian patients with clinical stage 1A NSCLC, surgical resection was associated with significantly better long-term OS than was radiotherapy. Five-year OS was 37.8% for surgery versus 20.7% for radiation in the propensity-matched analysis. These findings demonstrate that meaningful long-term survival is achievable in selected patients aged ≥ 90 years.

Our study expands on work by Yang et al.^7^, who analyzed outcomes in nonagenarians with NSCLC across all stages and treatments and reported that treatment of any kind conferred a survival advantage but remained underutilized. By focusing specifically on stage 1A disease and directly comparing surgery with radiation, our analysis provides more granular, contemporary insight into treatment selection in this population. This is further strengthened using propensity score matching and subgroup analyses across comorbidity burden and facility type.

Our results are consistent with earlier studies reporting improved observed survival among surgically treated patients, although these findings may reflect differences in patient selection.^11^ Likewise, Dong et al.^12^ found that, in patients aged ≥ 70 years, outcomes after surgery and SBRT were comparable, though a survival hazard favoring surgery remained. In our cohort, surgically treated nonagenarians frequently achieved multi-year survival, with nearly 11% surviving a decade after resection, comparable to reported outcomes in octogenarians.^5,10,13^

Subgroup analyses provide additional context. Although patients with higher CDS had lower absolute survival, the relative survival advantage associated with surgery persisted across comorbidity strata, aligning with prior literature suggesting that comorbidity burden alone should not preclude surgical consideration.^14–16^ Additionally, the subset of patients who refused surgery and received radiation alone had outcomes inferior to those who underwent resection, although these findings must be interpreted cautiously given the likelihood of residual selection bias and unmeasured differences in baseline health status.^17,18^

Prior work in octogenarians (age ≥ 80–85 years) has reported 5-year survival rates in the 40–60% range after surgical resection for early-stage NSCLC.^19,20^ Our findings show that, even among nonagenarians, long-term survival that many would consider clinically meaningful remains achievable. A few observations from our data have potential implications on clinical practice. First, prior to 12 months, radiation seems to possess a survival advantage over surgery. The survival curves intersect at roughly 12 months and then diverge thereafter. The 90-day perioperative mortality rate of nonagenarians undergoing surgery, at 9%, is markedly increased compared with the 1.1% operative mortality rate observed for all patients undergoing pulmonary resection in the Society of Thoracic Surgeons database.^21^ As such, operating on nonagenarians does pose significant risk, and preoperative counseling in this population should note the increased short-term risk but long-term benefit of surgery. Importantly, no a priori thresholds defining acceptable perioperative mortality risk or minimum survival benefit were established in this retrospective analysis, as treatment decisions reflected real-world clinical practice rather than protocolized selection criteria. The observed outcomes therefore should not be interpreted as supporting surgical intervention for all nonagenarians. Instead, these findings highlight the importance of individualized risk assessment, incorporation of patient goals and life expectancy, and shared decision-making when considering operative management in this population, where competing mortality risks are substantial. Second, although 5-year OS for nonagenarians in the surgical group, at 37.8%, was significantly better than (and nearly double) that of radiation, at 20.7%, younger patients who undergo resection for stage IA lung cancer typically experience a 70–90% survival rate.^22^ It is probable (though unproven by this study) that much of this difference relates to non-cancer causes of death. Furthermore, the outlook for a 90-year-old individual is quite different from that of a 95-year-old person, the latter of which is quite unlikely to survive to age 100 with or without a diagnosis of lung cancer.^23,24^ As such, we believe that the closer a patient approaches age 95 years, the less beneficial surgical treatment becomes. Performing surgical resection in a patient in their early 90s (especially age 90 or 91) with stage IA lung cancer can be justified, whereas operating on a 95- to 96-year-old individual may not be, as outcomes within the nonagenarian population are likely heterogeneous, and the balance between operative risk and potential long-term benefit may change with advancing age. Although these observations raise the possibility that treatment benefit could diminish among the oldest patients within this cohort, age-specific effects were not directly evaluated in the present study and should therefore be interpreted cautiously. Future analyses incorporating finer age stratification and geriatric assessment metrics are needed to better define treatment selection across the nonagenarian age spectrum.

Third, anatomic resection (lobectomy and segmentectomy) was associated with improved 5-year survival versus wedge resection, at roughly 45% compared with 30%. Indeed, the overall advantage of surgery versus radiation could potentially be attributed to patients undergoing anatomic resection, as the 30% 5-year survival after wedge resection is only slightly higher than the 23.5% after radiation in those who refused surgery. It is certainly possible, though, and even likely, that at least part of the explanation for the differences in survival between anatomic resection and wedge resection is that patients undergoing anatomic resection were healthier and fitter than those undergoing wedge resection. Given this doubt, it remains unclear whether or not to recommend lobectomy over wedge resection in a nonagenarian with a tumor that would be amenable to the latter. However, our data would suggest that, for a patient in their early 90s and with a central tumor, lobectomy would be a very reasonable option and likely to confer survival benefit over radiation.

Our study has several limitations. As a retrospective registry-based analysis, selection bias remains possible despite propensity score matching. The NCDB lacks key clinical variables that influence treatment decision-making in older adults, including performance status, frailty indices, pulmonary function testing, functional reserve, and cause-specific survival. Because physiologic fitness and frailty likely play a dominant role in treatment selection among nonagenarians, the magnitude of residual confounding may be substantial despite statistical adjustment, and observed survival differences should therefore be interpreted cautiously. Immortal time bias may also influence comparisons between treatment groups in observational analyses. The NCDB does not provide reliable data on time from diagnosis to treatment, so it is difficult to ascertain whether or not surgical patients experienced a delay compared with radiation patients that would have caused immortal time bias to favor survival in the surgical group. Importantly, the assumption that SBRT is consistently delivered earlier than surgery is not well established. Analyses comparing early SBRT with delayed surgery have noted that the optimal timing of these treatments remains unclear and that extended delays before surgery may still be associated with favorable outcomes.^25^ Therefore, the direction of immortal time bias in such comparisons cannot be definitively determined. Radiation parameters such as dose, fractionation, and technique are not captured, limiting treatment-level granularity. Although prior studies comparing surgery and SBRT in selected early-stage NSCLC populations have reported mixed results, with some analyses suggesting comparable or inferior long-term outcomes following SBRT in operable patients, the present study cannot directly evaluate these differences given the lack of treatment-specific radiation detail within the NCDB.^12^ Accordingly, observed survival differences between treatment groups should be interpreted as associative rather than causal.

In addition, OS may represent an incomplete measure of treatment value in this population. For many patients, preservation of functional independence, symptom control, recovery time, and quality of life may be equally or more important than longevity alone. Patient-reported outcomes and quality-of-life measures are not captured within the NCDB, limiting assessment of how different treatment strategies affect functional recovery and treatment-related burden. These limitations highlight opportunities for future investigation incorporating geriatric assessment tools and patient-centered outcomes to better inform shared decision-making in this population. Comparative effectiveness studies incorporating modern SBRT techniques alongside detailed frailty metrics may further refine treatment selection. Finally, the role of patient preferences and shared decision-making warrants continued exploration given the unique priorities of very elderly adults.

In conclusion, this national analysis represents the first study dedicated to comparing surgery and radiotherapy for stage 1A NSCLC in nonagenarians. Surgical resection was associated with higher observed long-term OS, but this association may reflect differences in patient selection that cannot be fully accounted for in registry data. For carefully selected patients aged ≥90 years, surgery remains a valid curative-intent option. Chronological age alone should not exclude these patients from multidisciplinary evaluation, particularly at centers equipped with geriatric assessment and minimally invasive thoracic surgical expertise.

## Supplementary Information

Below is the link to the electronic supplementary material.

Supplementary file1 (DOCX 914 KB)
