# Supplementary material for: Surgery Versus Radiation for Stage 1A NSCLC in Nonagenarians: 20 Years of Data, Decisions, and Outcomes
Source: Ann Surg Oncol. 2026 Apr 1;33(7):6252–62. doi: 10.1245/s10434-026-19551-y (PMC13242388; doi:10.1245/s10434-026-19551-y)

# **Supplementary Figure 1**: Overall survival by surgical resection type among Stage 1A NSCLC patients


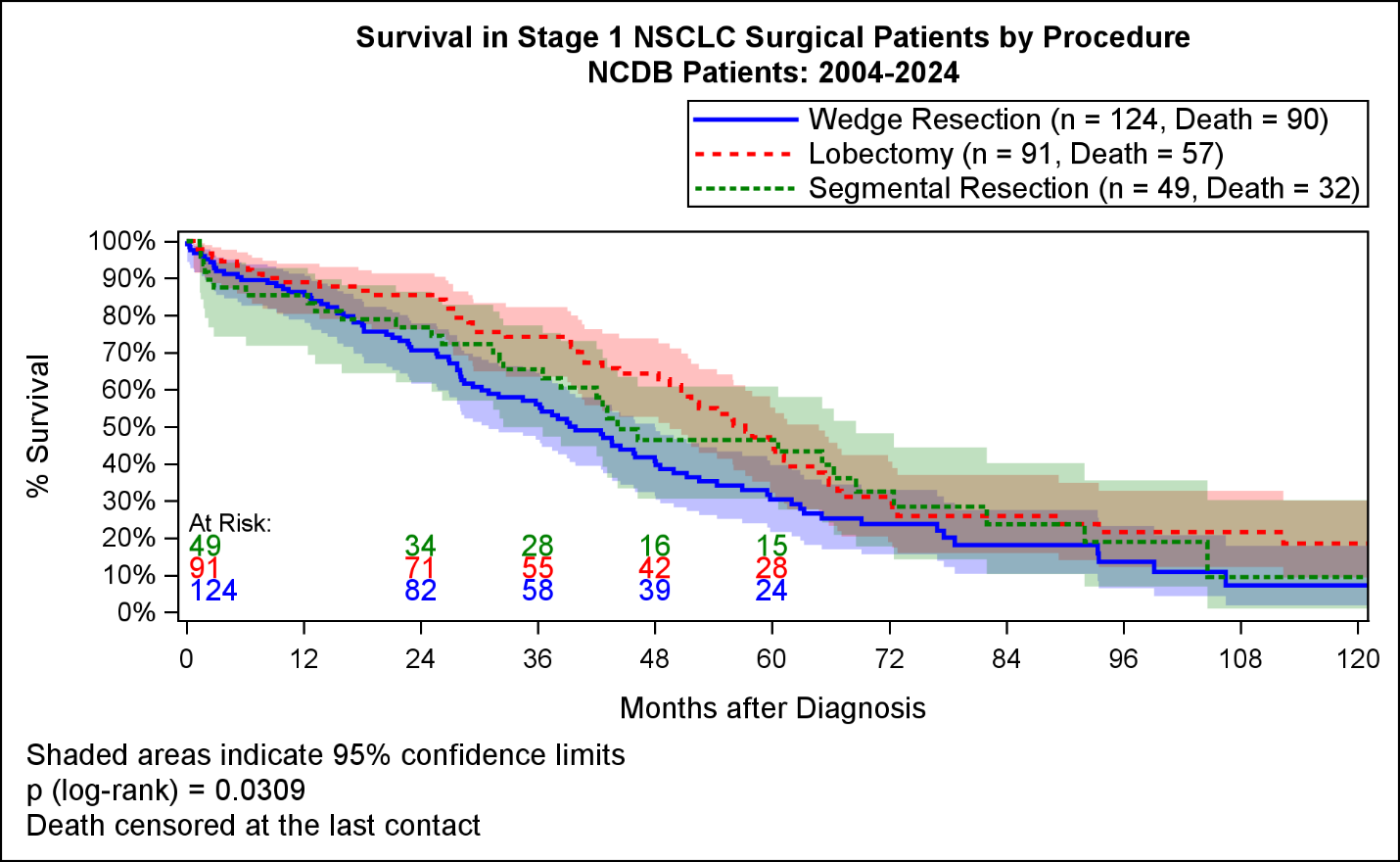


**Supplementary Figure 2.** Overall survival among surgically treated nonagenarian patients (≥ 90 years) with Stage 1A NSCLC stratified by Charlson–Deyo Comorbidity Score (CDS)


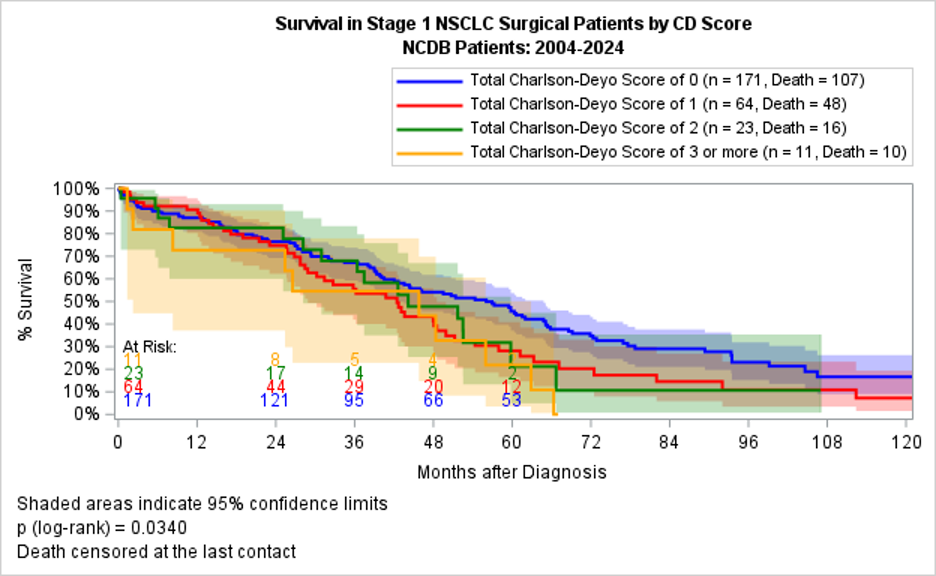


**Supplementary Figure 3.** Overall survival among radiation treated nonagenarian patients (≥ 90 years) with Stage 1A NSCLC stratified by Charlson–Deyo Comorbidity Score (CDS)


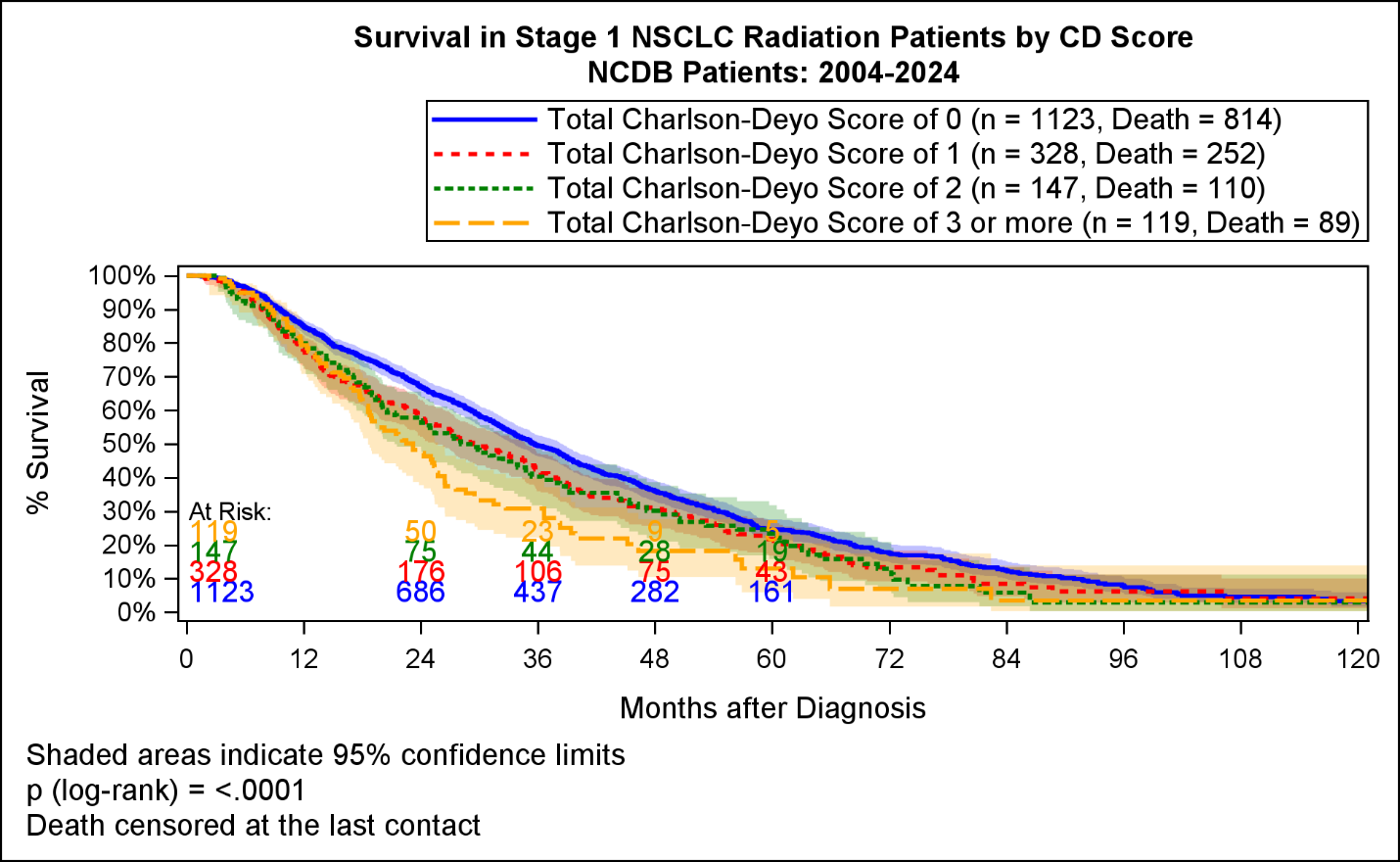


**Supplementary Figure 4.** Overall survival among surgically treated nonagenarian patients (≥90 years) with Stage 1A NSCLC by facility type
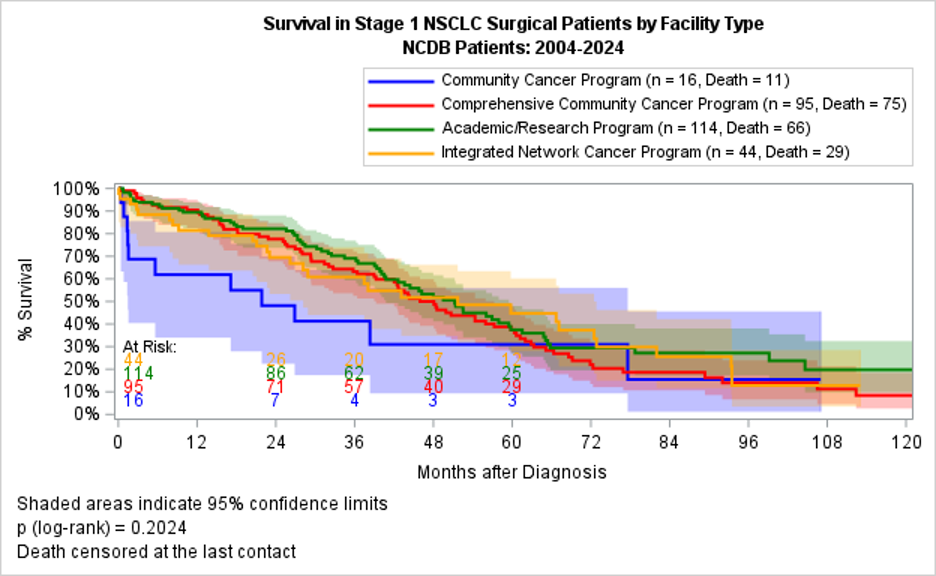


**Supplementary Figure 5.** Overall survival among radiation treated nonagenarian patients (≥90 years) with Stage 1A NSCLC by facility type


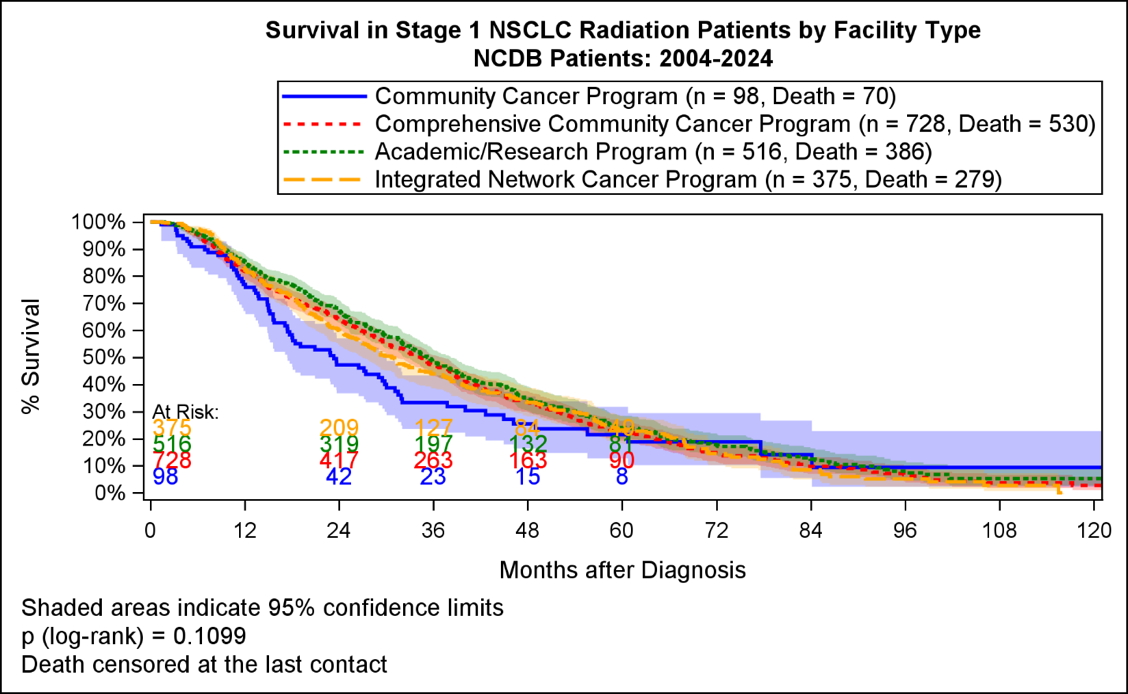

Supplement: Supplementary file 1 — Supplementary file1 (DOCX 914 KB) [file 10434_2026_19551_MOESM1_ESM.docx]
